# Immunogenic Biomarkers HMGB1 and sRAGE Are Potential Diagnostic Tools for Ovarian Malignancies

**DOI:** 10.3390/cancers15205081

**Published:** 2023-10-20

**Authors:** Lars Schröder, Alexander B. A. Rupp, Kathrin M. E. Gihr, Makbule Kobilay, Christian M. Domroese, Michael R. Mallmann, Stefan Holdenrieder

**Affiliations:** 1 Department of Obstetrics and Gynecology, University Hospital Cologne, 50931 Cologne, Germany; 2 Department of Obstetrics and Gynecology, Ketteler Hospital, 63071 Offenbach, Germany; 3 Institute of Clinical Chemistry and Clinical Pharmacology, University Hospital Bonn, 53127 Bonn, Germany; 4 Munich Biomarker Research Center, Institute of Laboratory Medicine, German Heart Centre, Technical University Munich, 80636 Munich, Germany; 5 CEBIO GmbH—Center for Evaluation of Biomarkers, 81679 Munich, Germany

**Keywords:** HMGB1, RAGE, PD1, PD-L1, ovarian cancer, diagnosis

## Abstract

**Simple Summary:**

Ovarian cancer is often diagnosed only in advanced stages, which limits therapeutic options and prognosis. Therefore, better and easily accessible methods for the early diagnosis of ovarian cancer are needed. In particular, blood-based biomarkers seem to be promising candidates for the accurate detection of ovarian cancer. We determined the concentrations of the two proteins HMGB1 and sRAGE in the sera of 231 women with ovarian cancer, benign diseases and without known gynecologic disease. In the analyses of receiver operating characteristics, both HMGB1 and sRAGE could distinguish patients with ovarian cancer from healthy women (area under the curve (AUC) 0.77 and 0.65), benign diseases (AUC 0.72 and 0.61) or all non-malignant cases (AUC 0.74 and 0.63). Moreover, the ratio of HMGB1/sRAGE differentiated even better between malignancies and other cases (AUC 0.78, 0.74 and 0.76, respectively). In conclusion, HMGB1 and sRAGE are potential candidates for the development of assays for early diagnosis of ovarian cancer and warrant inclusion in further validation studies.

**Abstract:**

Background: High mobility group box 1 (HMGB1), soluble receptor of advanced glycation end products (sRAGE) and programmed cell death markers PD-1 and PD-L1 are immunogenic serum biomarkers that may serve as novel diagnostic tools for cancer diagnosis. Methods: We investigated the four markers in sera of 231 women, among them 76 with ovarian cancer, 87 with benign diseases and 68 healthy controls, using enzyme immunoassays. Discrimination between groups was calculated using receiver operating characteristic (ROC) curves and sensitivities at fixed 90% and 95% specificities. Results: HMGB1 levels were significantly elevated and sRAGE levels were decreased in cancer patients as compared to benign and healthy controls. In consequence, the ratio of HMGB1 and sRAGE discriminated best between diagnostic groups. The areas under the curve (AUCs) of the ROC curves for differentiation of cancer vs. healthy were 0.77 for HMGB1, 0.65 for sRAGE and 0.78 for the HMGB1/sRAGE ratio, and slightly lower for the differentiation of cancer vs. benigns with 0.72 for HMGB1, 0.61 for sRAGE and 0.74 for the ratio of both. The highest sensitivities for cancer detection at 90% specificity versus benign diseases were achieved using HMGB1 with 41.3% and the HMGB1/sRAGE ratio with 39.2%, followed by sRAGE with 18.9%. PD-1 showed only minor and PD-L1 no power for discrimination between ovarian cancer and benign diseases. Conclusion: HMGB1 and sRAGE have differential diagnostic potential for ovarian cancer detection and warrant inclusion in further validation studies.

## 1. Introduction

Immunologic biomarkers are promising candidates for the early detection of malignant diseases, as well as for prognosis and monitoring of therapeutic success in a variety of malignancies including ovarian cancer [1,2,3,4,5]. In this respect, high-mobility group box 1 protein (HMGB1) and receptor for advanced glycation end products (RAGE) are two potential tumor markers capable of improving the diagnosis and treatment of ovarian cancer. Beside its role in regulation of transcription and chromatin remodeling, HMGB1 acts as a cytokine. It is synthesized actively and released both actively and passively by a variety of cells. These secretion processes predominantly take place in damaged or impaired cells, inflammatory environments and highly proliferating tumor tissue, although HMGB1 is present in a variety of physiologically occurring tissues [6,7]. HMGB1 can therefore be imputed to damage-associated molecular patterns (DAMPs). It was shown that HMGB1 may be a future marker in hormone-related cancer including ovarian cancer [8]. In a study by Li and Wei, the expression of HMGB1, together with the expression of BRCA1 and P62, was associated with drug resistance and sensitivity to chemotherapy in ovarian cancer [9]. As part of a prognostic model based on differentially expressed genes, HMGB1 showed promising results in the prognosis of ovarian cancer [10]. HMGB1 can be bound by RAGE, which exists in membrane-bound and soluble forms. Soluble RAGE (sRAGE) is deemed to intercept circulating HMGB1, thus limiting its effect [6]. Hence, sRAGE is depleted by increasing concentrations of HMGB1. Both HMGB1 and sRAGE have been studied concerning their application in ovarian cancer, yet results have not been conclusive so far [11,12,13].

Programmed cell death protein 1 (PD1) and one of its ligands, programmed cell death 1 ligand 1 (PD-L1), are involved in immunosuppression. Increased expression of PD-L1 in tumor tissue results, for instance, in resistance to lysis mediated by cytotoxic T cells, apoptosis of T cells and induction of inhibitory regulatory T cells [14]. Hence, the PD1/PD-L1 system is of great interest concerning the development of immune checkpoint inhibition therapies in various types of cancer [15]. However, it is still under debate to which extent measurements of concentrations of PD1 and PD-L1 alone can be used to predict malignancies, therapy response or survival of patients [16].

In this study, concentrations of the immunogenic biomarkers HMGB1, sRAGE, PD1 and PD-L1 were determined in the blood samples of 231 women with ovarian cancer, inflammation, cysts, other benign diseases or without any diagnosed severe disease. Additionally, concentrations of the established biomarkers CA-125 and tissue polypeptide antigen (TPA) were used for comparison.

## 2. Materials and Methods

### 2.1. Study Samples

All participants were recruited at the Clinic of Gynecology and Obstetrics of the University Hospital Bonn (Bonn, Germany). Serum samples were drawn between 2009 and 2015. Collection of samples was performed in the course of routine sample collection for the Biobank at the Institute of Clinical Chemistry and Clinical Pharmacology (University Hospital Bonn). Samples were consecutively collected before the start of therapy after cubital venipuncture in gel serum tubes (Sarstedt, Nurmbrecht, Germany). After drawing, samples were transferred to the central lab and centrifuged at room temperature for 10 min at 3220× *g*, and serum was aliquoted and stored at −80 °C in the Biobank until the time of measurement.

Patients were eligible for participation in the malignancy group if they suffered from ovarian cancer. Patients with ovarian cysts, inflammatory diseases of the ovaries or Fallopian tubes and patients with benign ovarian tumors were included as the benign group. Absence of known gynecologic diseases or malignancies was required for female individuals to be included in the group of healthy controls. Participation in the study was denied if another severe internal illness such as chronic kidney disease, diabetes, former malignant diagnoses or HIV infection was present.

### 2.2. Laboratory Assays

Concentrations of HMGB1 and sRAGE biomarkers were determined by use of the commercially available HMGB1 enzyme-linked immunosorbent assay (ELISA, IBL International, Hamburg, Germany) and the human RAGE Quantikine^®^ ELISA (R&D Systems, Minneapolis, MN, USA) according to the instructions of the manufacturers. The assays have been validated earlier [17,18]. PD-L1 and PD1 were measured with lab-developed and validated assays as described in Kruger et al. [19].

### 2.3. Statistics

IBM SPSS Statistics Version 23 software package (IBM, Armonk, NY, USA) was used for statistical data analyses. A significance level of α = 0.05 was used for assessment of statistical significance in all analyses. Mann–Whitney U tests and Kruskal–Wallis tests were applied in order to assess differences between 2 or more groups, respectively. Correlations between concentrations of HMGB1 and sRAGE were calculated with Spearman correlation coefficients. Receiver operating characteristic (ROC) curve analyses including calculations of sensitivities at fixed specificities of 90% (Sens_90_) and 95% (Sens_95_) were performed so as to investigate the diagnostic ability of the respective biomarkers. In case of sRAGE, lower values were used to indicate more positive results in ROC curve analyses.

## 3. Results

In total, 231 women were included in the study. Of these, 68 (30%) were healthy controls, 26 (11%) had ovarian cysts, 3 (1%) had an inflammatory disease of the ovaries or Fallopian tubes, 58 (25%) had benign tumors of the ovaries or Fallopian tubes and 76 (33%) were diagnosed with ovarian cancer. Of the women with ovarian cancer, 51 women (67%) had a primary tumor, while the remaining 25 (33%) suffered from a recurrent malignancy. Furthermore, 5 malignancies (7%) were staged as FIGO I, 2 (3%) as FIGO II, 46 (60%) as FIGO III and 3 (4%) as FIGO IV. For the remaining 20 malignant tumors (26%), no information regarding FIGO stages was available. Forty-eight (63%) of the malignant tumors were histologically classified as serous carcinomas, whereas ten (13%) were of non-serous histology. In 18 cases (24%), no histological classification was available.

**Figure 1:**
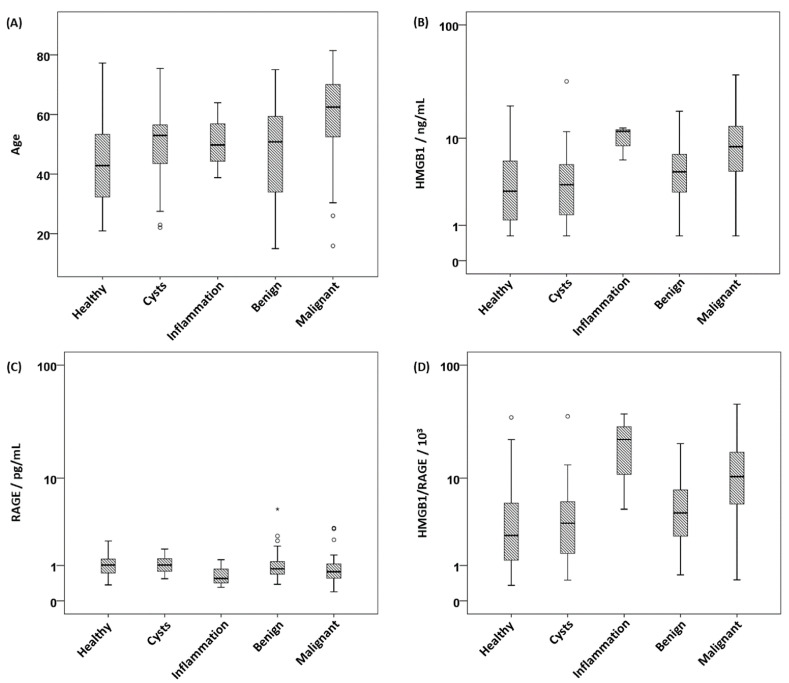
Boxplots of (**A**) age, (**B**) concentrations of HMGB1, (**C**) concentrations of sRAGE and (**D**) ratios of HMGB1 and sRAGE. Box indicates values between the 25th and 75th percentile, circles indicate outliers and asterisks indicate extreme outliers.

As shown in Table 1, the five subgroups differed significantly in age (*p* < 0.001) as well as serum concentrations of HMGB1 (*p* < 0.001), sRAGE (*p* = 0.006) and the HMGB1/sRAGE ratio (*p* < 0.001). All differences survived Bonferroni correction for multiple testing. Healthy controls showed the lowest median age, the lowest median HMGB1 concentration and, together with the group of ovarian cysts, the highest median sRAGE concentration. Accordingly, the HMGB1/sRAGE ratio was also lowest in the group of healthy controls. Women with an inflammatory disease had the highest median values in terms of HMGB1 and the HMGB1/sRAGE ratio as well as the lowest sRAGE levels. Median sRAGE concentration in the malignancy group was the second lowest, while HMGB1 and the HMGB1/sRAGE ratio were the second highest of all groups. In addition, malignant cases possessed the highest median age. No significant differences in serum concentrations of PD1 or PD-L1 were found. The respective boxplots are shown in Figure 1.

Correlations between concentrations of HMGB1 and sRAGE yielded negative correlation coefficients when only malignant cases, only benign cases, only healthy controls or all participants were considered. However, only the respective correlation comprising all participants was significant (ρ = −0.220, *p* = 0.001).

When cancer patients with primary tumors were compared to those with recurrent tumors, primary tumors exhibited significantly higher concentrations of HMGB1 (*p* < 0.001) and a higher HMGB1/sRAGE ratio (*p* < 0.001) than recurrent tumors, as given in Table 2. Initially significantly lower concentrations of sRAGE (*p* = 0.017) in primary tumors failed to remain significant after Bonferroni correction. Both groups did not differ significantly in age, PD1 and PD-L1.

The results from the ROC curve analyses are shown in Table 3. HMGB1 showed consistently high AUCs of above 0.7 in the discrimination of malignant cases from healthy controls (AUC = 0.771, *p* < 0.001), benign cases (AUC = 0.722, *p* < 0.001) or all non-malignant cases (AUC = 0.743, *p* < 0.001). Discrimination between healthy and benign cases yielded a lower AUC of 0.605 (*p* = 0.026). sRAGE was also able to differentiate significantly between malignancies and healthy controls (AUC = 0.650, *p* = 0.002), benign diseases (AUC = 0.612, *p* = 0.014) or all non-malignant cases (AUC = 0.628, *p* = 0.002), yet with somewhat lower AUCs compared to the results of HMGB1. A respective differentiation between healthy and benign cases was not significant (*p* = 0.353). Using the ratio HMGB1/sRAGE resulted in slightly higher AUCs compared to HMGB1 and also allowed for a significant distinction of malignancies from healthy controls (AUC = 0.782, *p* < 0.001), benign cases (AUC = 0.737, *p* < 0.001) or all non-malignant cases (AUC = 0.757, *p* < 0.001). As with HMGB1, differentiation between healthy and benign participants was also significant (*p* = 0.018) but led to a lower AUC of 0.611.

**Figure 2:**
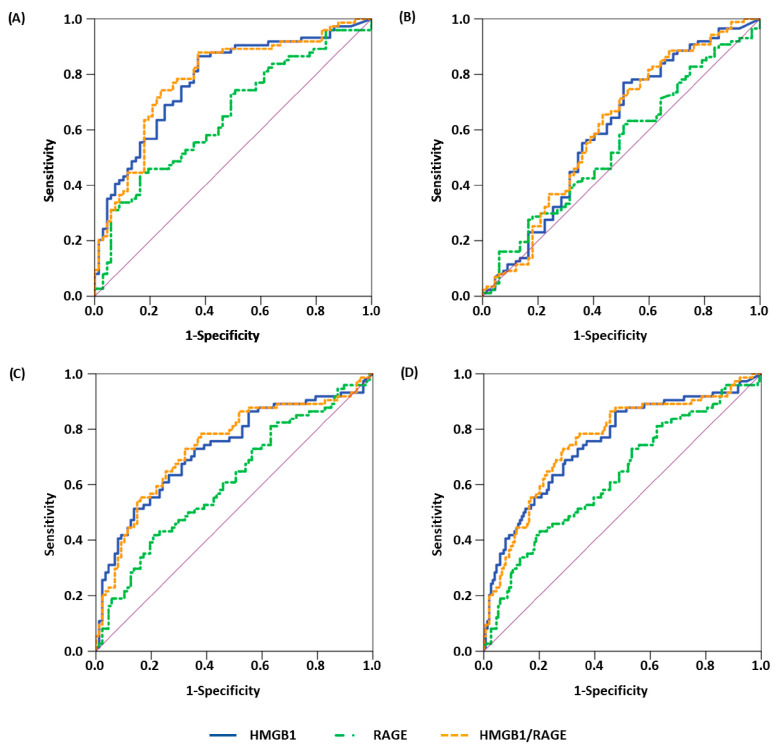
ROC curves of HMGB1 (blue, continuous), sRAGE (green, dashed-dotted) and HMGB1/sRAGE (orange, dashed) ratio for differentiation between (**A**) healthy and malignant cases, (**B**) healthy and benign cases, (**C**) benign and malignant cases and (**D**) all non-malignant and malignant cases. Purple lines represent lines of no-discrimination.

Similar results were obtained for sensitivities at fixed specificities of 90% (Sens_90_) and 95% (Sens_95_), with HMGB1 and HMGB1/sRAGE showing the highest Sens_90_, between 36.5% and 41.3%, as well as the highest Sens_95_, between 23.0% and 34.7%, for ROCs with malignancies as classifier, while respective values of Sens_90_ (9.2–11.5%) and Sens_95_ (6.9%) were lower in the differentiation between healthy and benign cases. sRAGE, in general, showed lower sensitivities when malignant diagnosis was used as classifier, with Sens_90_ between 18.9% and 33.8% and Sens_95_ between 12.2% and 16.2%. ROC curve analyses with healthy and benign cases yielded similar sensitivities as with HMGB1 or HMGB1/sRAGE (Sens_90_: 16.1%, Sens_95_: 4.6%). The ROC curves of HMGB1, sRAGE and the HMGB1/sRAGE ratio are depicted in Figure 2.

PD1 failed to distinguish between healthy and malignant or healthy and benign cases yet differentiated significantly between benign and malignant (AUC = 0.599, *p* = 0.029) as well as non-malignant and malignant cases (AUC = 0.594, *p* = 0.020). ROC curve analyses of PD-L1 yielded only non-significant results for differentiation between all respective subgroups. In addition, sensitivities were well below those of HMGB1, sRAGE or HMGB1/sRAGE and reached maximal values of 11.8% (Sens_90_) and 9.3% (Sens_95_).

## 4. Discussion

With an age-standardized incidence of 5.7–7 per 100,000 person years depending on the Human Development Index, ovarian cancer is a severe global health burden and the eighth most common cause of cancer death in women [20]. Despite the increasing availability of diagnostic resources, most cases of ovarian cancer still are only diagnosed at advanced stages, i.e., FIGO stages III or IV, and therefore are associated with poor prognosis [21]. A variety of potential biomarkers has been examined for the detection of ovarian cancer including proteins, miRNAs, ctDNA, methylation patterns and transcriptomics [22,23,24,25,26]. In particular, cancer antigen 125 (CA-125) has been studied and is applied as a biomarker for the monitoring of treatment response in ovarian cancer [27,28]. However, due to its rather low sensitivity for early stages of ovarian cancer, CA-125 is not considered an appropriate screening tool [27], although a two-step screening model comprising CA-125 and transvaginal ultrasound has been proposed by the Normal Risk Ovarian Screening Study [29]. Similar issues arise also with other biomarkers such as HE4 [30]. Additionally, certain other problems may occur in the application of biomarkers, for instance, an influence of other, benign conditions on biomarker concentrations. Therefore, there is still a need for new biomarkers in the detection of ovarian cancer as well as for prognosis.

In this study, the immunogenic and inflammatory proteins HMGB1, sRAGE, PD1 and PD-L1 were investigated as possible biomarkers in ovarian cancer. Apart from the three patients with inflammatory disease, women with malignant tumors had the highest concentration of HMGB1 and also the highest HMGB1/sRAGE ratios. The concentration of sRAGE, on the other hand, was lower in the malignant group than in healthy controls, ovarian cysts or other benign diseases. These results correspond with the known role of HMGB1 or sRAGE and with initial expectations. Both HMGB1 and sRAGE are involved in inflammation and in metabolism within the microenvironment of malignant tumors [31,32,33]. Therefore, it is reasonable to anticipate the highest levels of HMGB1 in these two subgroups. As soluble RAGE is able to function as a decoy receptor for HMGB1, its concentration decreases in the presence of higher amounts of HMGB1. Taken together, the HMGB1/sRAGE ratio should increase in patients with malignant or inflammatory diagnosis, as is shown in our data. It is important to distinguish measurements of serum concentrations of soluble RAGE from quantification of RAGE in tissue samples. An overexpression of RAGE has been found in a study with ovarian cancer tissue samples of 25 women, for instance [34]. However, expression of RAGE in tumor tissue may be elevated so as to counterbalance the increased consumption due to elevated levels of HMGB1, but still lead to decreased concentrations of free, soluble RAGE in serum.

Differences between primary and recurrent ovarian cancer can be well explained by the distribution of FIGO stages. The initial diagnosis of ovarian cancer often occurs in FIGO stages III or IV due to the lack of early symptoms in localized stages, hence resulting in a relatively large tumor mass at the time of diagnosis and a consistently higher secretion of HMGB1 with subsequent depletion of sRAGE as described above. Owing to follow-up care programs, recurrent malignancies may already be detected when the total tumor mass is still small—although the respective FIGO stage may still be high because of the localization of recurrent tumors. In accordance with these considerations, primary tumors showed higher amounts of HMGB1, lower levels of sRAGE and a higher HMGB1/sRAGE ratio than recurrent malignancies.

In the ROC curve analyses, HMGB1 and the HMGB1/sRAGE ratio showed comparably good results in terms of AUCs or sensitivities at specificities of 90% or 95% and were superior compared to sRAGE. Both HMGB1 and the HMGB1/sRAGE ratio were not only able to distinguish healthy controls from malignant cases but also achieved significant differentiation between benign and malignant diagnosis. Although this study lacks validation in an independent cohort, HMGB1 and the HMGB1/sRAGE ratio clearly seem to be promising and easily accessible biomarkers for the detection of ovarian cancer and possibly even for benign ovarian diseases. It is of course necessary to confirm these results in consecutive studies with a prospective design and comprehensive collection of possibly confounding baseline characteristics, yet even with these constraints, the present results justify further investigation towards the applicability of HMGB1 and the HMGB1/sRAGE ratio as a diagnostic tool in ovarian cancer. To date, only a few other studies have examined HMGB1 in the diagnosis or prognosis of ovarian cancer, and blood-based studies are scarce. In several studies, HMGB1 expression in tissue samples of ovarian cancer was predictive for poor survival of patients with ovarian cancer [12,35]. Another report about measurement of HMGB1 concentrations in the serum samples of 105 patients with ovarian cancer, 46 patients with benign ovarian disease and 33 healthy controls is consistent with our results and showed significantly elevated HMGB1 levels in malignant cases, yet no ROCs or sensitivities were reported [36]. Therefore, results from ROC curve analyses can only be compared to those of other biomarkers. In a parallel publication, we investigated CA-125 and tissue peptide antigen (TPA) in a sample of patients with malignant or benign ovarian or uterine diagnoses [37]. The samples of this study were included in the parallel publication. Both CA-125 and TPA yielded even higher AUCs of >0.8 and higher sensitivities at specificities of 90% or 95%. Consequently, it is likely that HMGB1 or the HMGB1/sRAGE ratio alone will not outperform these established tumor markers. However, CA-125 and TPA tended to show lower AUCs and sensitivities in the detection of ovarian cancer in early stages. Therefore, the combination of these established biomarkers with HMGB1 and/or the HMGB1/sRAGE ratio may improve such detection, thereby leading to earlier diagnosis and resulting in better prognosis for patients with ovarian cancer. The combinations of several different biomarkers with CA-125 have been reported, yet no combination was able to improve the diagnostic capability compared to CA-125 alone [24]. In this respect, more studies with a prospective design and a variety of clinical characteristics will be needed to further evaluate possible applicability of HMGB1, sRAGE and the HMGB1/sRAGE ratio in ovarian cancer.

Despite these promising results, certain limitations of the present study must be considered. First, this study is limited by the usual restraints of retrospective studies and the lack of group matching. Moreover, only a few clinical characteristics were available. In consecutive, prospective studies, more baseline characteristics including long-term medication, smoking status, alcohol and substance abuse need to be collected so as to exclude confounding through these parameters. For instance, HMGB1 release has been shown to be influenced by cigarette smoke in lung tissue [37]. In addition, the significant difference in age between the malignant, benign and healthy subgroups may have confounded biomarker levels as well. So far, it is not fully understood whether physiological changes of HMGB1 and sRAGE occur at all during aging and if such changes are tissue-dependent. Levels of HMGB1 were found to decrease in neurons but increase in astrocytes during aging in mouse brains [38]. Further, a study with rat adipose tissue found evidence for a potential decrease in sRAGE with age [39]. Lastly, studies with a higher number of ovarian cancer cases in early stages will be needed to evaluate the potential of HMGB1 in the early detection of ovarian cancer, as HMGB1 expression in ovarian cancer tissue has been reported to increase in advanced stages [40,41]. However, as this study was intended as an exploratory approach to evaluate a possible use of HMGB1 and sRAGE as diagnostic tools in ovarian cancer, no prospective or matched design was used. It is clear that this difference in age needs to be avoided in future studies in order to get a definite answer to the question of if these biomarkers can reliably be applied in screening, diagnosis, monitoring or prognosis of ovarian cancer. On the other hand, it has to be pointed out that the cohort reflects the patients treated in a University Hospital setting. Sample collection, preanalytical handling and storage were carried out in a well standardized manner, lab analyses were quality-controlled and statistics were performed independently from sample collection and lab analyses.

## 5. Conclusions

High serum HMGB1 concentrations and low serum sRAGE levels were associated with ovarian cancer as compared to benign cases and healthy controls. The ratio of both markers, HMGB1/sRAGE, differentiated even better between women with ovarian cancer and healthy or benign controls. Future validation studies are warranted to confirm the potential applicability of HMGB1 and sRAGE in ovarian cancer diagnostics. To the best of our knowledge, this is also the first study to investigate the diagnostic ability of sRAGE and the HMGB1/sRAGE ratio in ovarian cancer.

## Figures and Tables

**Table 1:** Age and biomarker concentrations according to health status.

Parameter	Age ^1^	HMGB1 ^2^	sRAGE ^3^	HMGB1/sRAGE ^4^	PD-1 ^5^	PD-L1 ^6^
Healthy	43 (21)	2.90 (5.25)	1.02 (0.56)	2.59 (4.57)	0.011 (0.068)	0.1 (7.7)
N	68	67	67	67	66	67
Cysts	49 (17)	3.43 (4.16)	1.02 (0.52)	3.56 (4.71)	0.006 (0.041)	0.1 (9.0)
N	25	26	26	26	26	26
Inflammation	50 (−)	11.56 (−)	0.55 (−)	22.47 (−)	0.027 (−)	0.1 (−)
N	3	3	3	3	3	3
Ovarian benign	51 (27)	4.69 (4.29)	0.88 (0.48)	4.57 (5.31)	0.016 (0.058)	0.1 (7.4)
N	58	58	58	58	58	58
Ovarian cancer	62 (18)	8.33 (8.26)	0.77 (0.50)	10.35 (11.71)	0.023 (0.039)	0.1 (5.7)
N	76	75	74	74	76	75
*p*-value *	<0.001	<0.001	0.006	<0.001	0.152	0.824

Data are given as median (interquartile range). * Results from Kruskal–Wallis tests. ^1^ years; ^2^ data given in ng∙mL^−1^; ^3^ data given in pg∙mL^−1^, ^4^ data given in 10^3^, ^5^ data given in ng/mL, ^6^ data given in pg/mL.

**Table 2:** Age and biomarker concentrations of patients with primary and recurrent malignant tumors.

Malignancy	Age ^1^	HMGB1 ^2^	sRAGE ^3^	HMGB1/sRAGE ^4^	PD1 ^5^	PD-L1 ^6^
Primary	62 (20)	10.92 (9.67)	0.69 (0.39)	12.97 (16.80)	0.019 (0.039)	0.1 (2.2)
N	51	51	50	50	51	50
Recurrent	63 (18)	5.48 (5.21)	1.03 (0.59)	4.93 (9.43)	0.030 (0.040)	0.1 (9.0)
N	25	24	24	24	25	25
*p*-value *	0.462	<0.001	0.017	<0.001	0.436	0.484

Data are given as median (interquartile range). * Results from Mann–Whitney U tests. ^1^ years; ^2^ data given in ng∙mL^−1^; ^3^ data given in pg∙mL^−1^, ^4^ data given in 10^3^, ^5^ data given in ng/mL, ^6^ data given in pg/mL.

**Table 3:** Data from analysis of ROC curves.

Comparison	Parameter	AUC	95% CI	*p*-Value	Sens_90_	Sens_95_
healthy vs. malignant	HMGB1	0.771	0.693–0.849	<0.001	41.3	34.7
	sRAGE ^a^	0.650	0.559–0.740	0.002	33.8	12.2
	HMGB1/sRAGE	0.782	0.705–0.859	<0.001	36.5	27.0
	PD1	0.588	0.490–0.686	0.070	6.6	5.3
	PD-L1	0.503	0.407–0.599	0.954	9.3	4.0
healthy vs. all benign	HMGB1	0.605	0.512–0.697	0.026	11.5	6.9
	sRAGE ^a^	0.544	0.452–0.636	0.353	16.1	4.6
	HMGB1/sRAGE	0.611	0.519–0.703	0.018	9.2	6.9
	PD1	0.506	0.411–0.600	0.905	5.7	2.3
	PD-L1	0.492	0.399–0.585	0.860	3.4	1.1
all benign vs. malignant	HMGB1	0.722	0.641–0.802	<0.001	41.3	30.7
	sRAGE ^a^	0.612	0.525–0.699	0.014	18.9	16.2
	HMGB1/sRAGE	0.737	0.658–0.817	<0.001	39.2	23.0
	PD1	0.599	0.511–0.687	0.029	11.8	6.6
	PD-L1	0.510	0.421–0.600	0.819	10.7	9.3
all non-malignant vs. malignant	HMGB1	0.743	0.672–0.814	<0.001	41.3	30.7
	sRAGE ^a^	0.628	0.550–0.707	0.002	28.4	12.2
	HMGB1/sRAGE	0.757	0.687–0.826	<0.001	37.8	23.0
	PD1	0.594	0.521–0.668	0.020	9.2	6.6
	PD-L1	0.507	0.428–0.587	0.862	10.7	8.0

Underlined diagnoses were used as classifiers. AUC = area under the curve; CI = confidence interval; Sens_90_ = percent sensitivity at 90% specificity; Sens_95_ = percent sensitivity at 95% specificity. ^a^ Lower values indicated more positive results.

## Data Availability

The data presented in this study are available on request from the corresponding author. The data are not publicly available due to reasons of data and privacy protection laws.
